# Strategies and challenges of toxicity reduction in nasopharyngeal carcinoma in the era of precision radiotherapy: from paradigm shift to integrated decision-making

**DOI:** 10.3389/fonc.2026.1788757

**Published:** 2026-07-16

**Authors:** Yun Zheng, Bin Li

**Affiliations:** 1 Liyang Hospital of Chinese Medicine, Changzhou, Jiangsu, China; 2 The First Affiliated Hospital of Zhejiang Chinese Medical University (Zhejiang Provincial Hospital of Chinese Medicine), Hangzhou, Zhejiang, China

**Keywords:** advanced radiotherapy techniques, nasopharyngeal carcinoma, precision radiotherapy, three-dimensional strategy framework, toxicity reduction strategies

## Abstract

In the era of intensity-modulated radiotherapy (IMRT) and volumetric modulated arc therapy (VMAT) for nasopharyngeal carcinoma (NPC), local control rates have improved significantly, with 5-year local control rates now exceeding 90% for locally advanced disease. This progress has shifted the treatment focus from “ensuring survival” to “optimizing quality of survival.” Treatment-related acute and chronic toxicities are now recognized as major constraints on patients’ long-term quality of life. This review systematically summarizes and synthesizes the latest evidence on toxicity reduction through a three-dimensional strategy: spatial precision sculpting, temporal sequential optimization, and energy technology escalation. We critically examine the underlying logic, clinical implementation challenges, and ongoing controversies associated with each dimension, and explore their interdependence in clinical decision-making. Although much of the key evidence originates from large-scale clinical trials in high-incidence regions, the resulting principles are increasingly shaping global practice. For example, international guidelines now recommend target volume reduction based on tumor response to induction chemotherapy. However, this approach depends heavily on precise imaging and consistent contouring. Other developments include sequential chemoradiotherapy and chemotherapy de-escalation combined with immunotherapy, which challenge traditional standards but also raise new questions about patient selection and toxicity profiles. Meanwhile, proton therapy offers physical advantages but still lacks Phase III survival evidence and faces cost-effectiveness debates. The review concludes that successful toxicity reduction will require an individualized, integrated decision-making system. This system should incorporate multidimensional biomarkers, standardized patient-reported outcomes (PROs), and global real-world data, potentially synthesized by artificial intelligence (AI)-driven platforms to navigate the complex trade-offs between efficacy and toxicity. The goal is to move beyond “disease cure” toward value-based medicine focused on holistic recovery.

## 1 Introduction: the paradigm shift from “cure” to “quality of survival” in global context

Nasopharyngeal carcinoma (NPC) has distinct epidemiological patterns: it is common in East and Southeast Asia, where it is closely linked to Epstein-Barr virus (EBV) infection, but rare in Western populations, where smoking and alcohol may play a greater role (1). Regardless of these differences, the global adoption of advanced delivery techniques, such as IMRT and VMAT, over the past two decades has dramatically improved outcomes, achieving 5-year local control rates over 90% for locally advanced disease (2). For most patients, “cure” is now a realistic goal.

**Figure 1:**
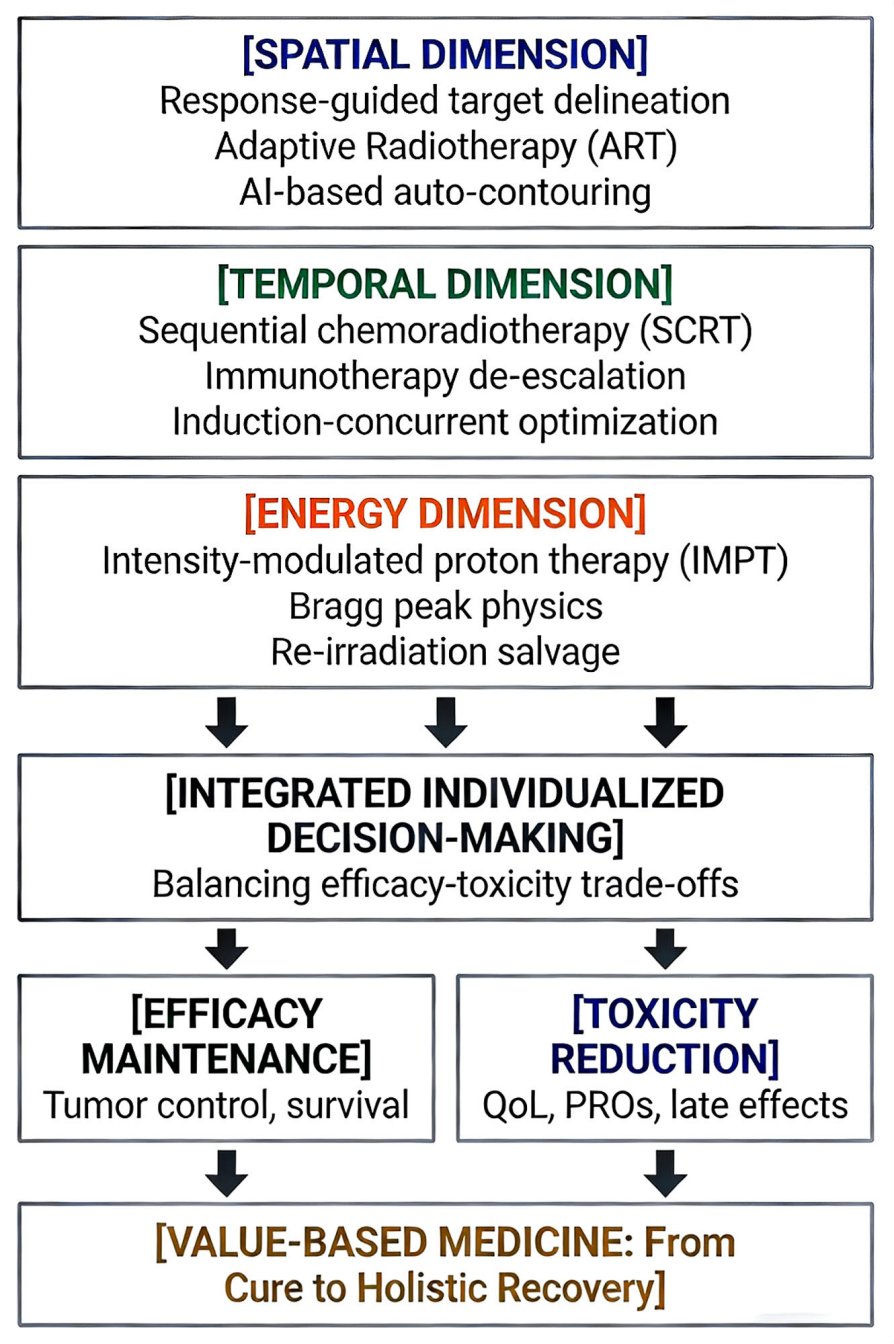
The three-dimensional framework for toxicity reduction in NPC in the precision radiotherapy era. This integrated framework combines spatial (precision target sculpting), temporal (sequential optimization), and energy (advanced technology escalation) dimensions. The convergence of these dimensions, guided by individualized decision-making, aims to maintain efficacy while reducing toxicity.

However, with longer survival, the long-term toxicities of chemoradiation have become increasingly evident (2). Severe permanent side effects—such as xerostomia, swallowing dysfunction, hearing loss, neck fibrosis, and radiation-induced brain injury—significantly impair patients’ physical function, nutrition, psychological health, and social reintegration (3). Consequently, a new global consensus is emerging: treatment goals must expand from the single aim of “cure” to also include “quality of survival.” In this new paradigm, toxicity reduction has risen from a secondary concern to a core treatment endpoint, equal in importance to efficacy enhancement (See **Figure 1**).

It is worth noting that high-incidence regions such as China have conducted large, high-quality randomized trials that provide leading evidence on toxicity reduction strategies (4). These studies help address a universal challenge in NPC care: how to reduce toxicity without compromising excellent tumor control.

This review summarizes key evidence supporting the three-dimensional toxicity reduction strategy, synthesizes the interplay between these dimensions, discusses their relevance to global practice, and highlights scientific questions and future directions that require worldwide collaboration.

## 2 Spatial dimension strategy: precision target sculpting — advances and uncertainties

### 2.1 Core concept: from “empirical margins” to “response-guided precision”

Traditional NPC radiotherapy used conservative target volumes based on pre-chemotherapy tumor extent, plus a uniform margin (usually 5–10 mm). This “one-size-fits-all” approach aimed to cover potential microscopic spread but resulted in unnecessary radiation to sensitive organs—a major cause of late toxicities.

In the precision radiotherapy era, the spatial strategy has shifted fundamentally: the target volume is now seen as a dynamic, biological entity that should be adjusted based on tumor response to induction chemotherapy and advanced imaging (5). The goal is to balance “delivering a lethal dose to the tumor” with “maximally protecting normal tissues,” reducing toxicity by limiting high-dose exposure at its source.

### 2.2 Key evidence-based advances

#### 2.2.1 Landmark phase III evidence

In 2025, a Phase III trial led by Prof. Ma Jun (Sun Yat-sen University Cancer Center) and published in CA: A Cancer Journal for Clinicians provided pivotal evidence (6). The study randomized 449 locally advanced NPC patients to either precision reduced-volume radiotherapy (based on post-induction chemotherapy tumor shrinkage) or traditional pre-chemotherapy volumes. After 38 months median follow-up, local-regional recurrence-free survival was similar between groups (94.3% *vs*. 90.5%; hazard ratio for reduced-volume group: 0.67 [95% CI 0.42–1.06] within a non-inferiority margin of 1.53), as was overall survival. Importantly, the reduced-volume group had a 40.2% lower incidence of Grade 3+ oral mucositis (34.0% *vs*. 56.9%) and significantly less severe hearing loss and xerostomia. This confirms that response-based target reduction is both safe and effective for toxicity reduction (see **Table 1**).

**Table 1:** Summary of key clinical trials supporting toxicity reduction strategies in nasopharyngeal carcinoma.

Strategy domain	Trial/lead author	Year	Phase	Design	Key efficacy outcome	Key toxicity/quality-of-life outcome	Ref.
Spatial (Response-guided volume reduction)	Tang LL, Ma J (CA Cancer J Clin)	2025	III	Multicenter, open-label, non-inferiority (n=449)	3-yr LRRFS: 94.3% (reduced-volume) *vs*. 90.5% (conventional); non-inferior	Grade ≥3 oral mucositis: 34.0% *vs*. 56.9%; lower hearing loss and xerostomia	(6)
Temporal (Sequential chemoradiotherapy, SCRT)	Hu CS, He XY (JAMA Oncol)	2024	III	Multicenter, non-inferiority (n=468)	3-yr FFS: 83.7% (SCRT) *vs*. 79.5% (IC+CCRT)	Grade ≥3 acute toxicity during RT: 34.8% *vs*. 58.6%; less mucositis, nausea/vomiting	(7)
Temporal (PD-1 + RT, chemotherapy de-escalation)	Chen SY, et al. (Cell Rep Med)	2023	II	Single-arm, toripalimab + RT (*de novo* metastatic NPC)	ORR 89.7%; median PFS 25.9 months	No grade ≥4 irAEs; significantly lower chemotherapy-related toxicity	(8)
Temporal (PD-1 + RT *vs*. CCRT)	Zhang X, et al. (IJROBP)	2025	II	Randomized (camrelizumab ± SBRT for recurrent/metastatic)	Improved PFS with camrelizumab + SBRT (HR 0.46)	Lower grade 3+ adverse events in SBRT + camrelizumab arm	(9)
Energy (Proton therapy *vs*. IMRT)	Ishikawa Y, et al. (J Radiat Res)	2025	Retrospective matched-pair	Real-world comparison (prostate cancer, but similar head/neck findings cited)	No survival difference (non-inferior)	Significantly lower acute toxicities (e.g., xerostomia, dysphagia) and better patient-reported QOL	(10)
Energy (Proton dosimetry)	Hung HM, et al. (Med Dosim)	2022	Dosimetric	Comparison of IMPT *vs*. IMRT for recurrent NPC	N/A (physical endpoint)	Mean dose reduction: parotids 30–50%, cochlea 40%, brainstem 35%	(11)

LRRFS, locoregional recurrence-free survival; FFS, failure-free survival; ORR, objective response rate; PFS, progression-free survival; HR, hazard ratio; IC, induction chemotherapy; CCRT, concurrent chemoradiotherapy; RT, radiotherapy; SBRT, stereotactic body radiotherapy; irAEs, immune-related adverse events; QOL, quality of life; IMPT, intensity-modulated proton therapy; IMRT, intensity-modulated radiotherapy.

#### 2.2.2 Global guideline integration

This evidence was quickly incorporated into international guidelines. In 2025, the International Consensus Guideline on Delineation of the Clinical Target Volumes at Different Dose Levels for Nasopharyngeal Carcinoma (jointly published by multiple international societies) recommended delineating target volumes based on post-induction chemotherapy extent, with individualized margins (12). This approach, building upon earlier anatomical studies (5), has established a new global standard for toxicity-reductive targeting, with studies confirming its efficacy in long-term outcomes (13).

#### 2.2.3 Adaptive radiotherapy and technological enablement

Translating precision targeting into practice relies on technologies like Adaptive Radiotherapy (ART)—repeated imaging during treatment to adjust plans based on tumor shrinkage (14). Although NPC-specific Phase III survival data are pending, multi-center studies show ART reduces dose to critical organs and improves patient-reported outcomes (15). Furthermore, artificial intelligence, particularly deep learning, is emerging as a powerful tool for accurate and consistent target volume delineation, addressing inter-observer variability (16). These technologies are crucial for realizing the full “toxicity reduction” potential of spatial precision, with ongoing research optimizing their application (17).

### 2.3 Summary, challenges, and implementation bottlenecks

Spatial precision is fundamental to toxicity reduction, yet its global implementation faces several challenges:

- Technical dependence and contouring variability — Success requires high-quality MRI, clinical expertise, and consistent contouring. Differences in interpreting “high-risk microscopic spread” across institutions can lead to inconsistent practice, diminishing potential benefits. AI-based auto-segmentation tools are being developed to mitigate this variability (16).
- Biological uncertainty of response-adapted therapy — Imaging response may not fully reflect microscopic residual disease, posing a risk of geographic miss when targets are reduced. Biomarkers such as radiomics and circulating EBV DNA are being explored to better monitor tumor burden and predict recurrence, aiming to guide target adaptation more biologically (18–20).
- Need for long-term data — While early and mid-term results are promising, long-term outcomes (10+ years), including very late toxicities and recurrence patterns, require ongoing global multi-center follow-up.

## 3 Temporal dimension strategy: optimizing treatment sequencing and its dual effects

### 3.1 Core concept: separating toxicity peaks by reordering treatments

Concurrent chemoradiotherapy (CCRT) has long been standard for locally advanced NPC but leads to toxicity superposition: radiation-induced local toxicities (mucositis, dermatitis) coincide with chemotherapy’s systemic side effects (nausea, myelosuppression) (21). This overlap reduces tolerance, increases treatment breaks, and impairs long-term quality of life. The temporal strategy restructures the sequence of systemic therapy (chemotherapy, immunotherapy) and radiotherapy. By separating toxicity peaks in time, it aims to maintain treatment intensity while improving tolerability.

### 3.2 Key evidence-based advances

#### 3.2.1 Sequential chemoradiotherapy model

A Phase III trial by Prof. Hu Chaosu and Prof. He Xiayun (Fudan University Shanghai Cancer Center), published in JAMA Oncology, compared SCRT (induction chemotherapy + radiotherapy alone + adjuvant chemotherapy) with traditional induction chemo followed by CCRT (7). Both groups had similar 3-year failure-free survival (83.7% *vs*. 79.5%). However, the SCRT group had significantly lower Grade 3+ acute toxicity during radiotherapy (34.8% *vs*. 58.6%), especially for mucositis, nausea, and vomiting. This model, evolving from earlier sequential approaches (22), offers a validated option for patients intolerant to concurrent chemotherapy (see **Table 1**).

#### 3.2.2 Chemotherapy de-escalation with immunotherapy

**Figure 2:**
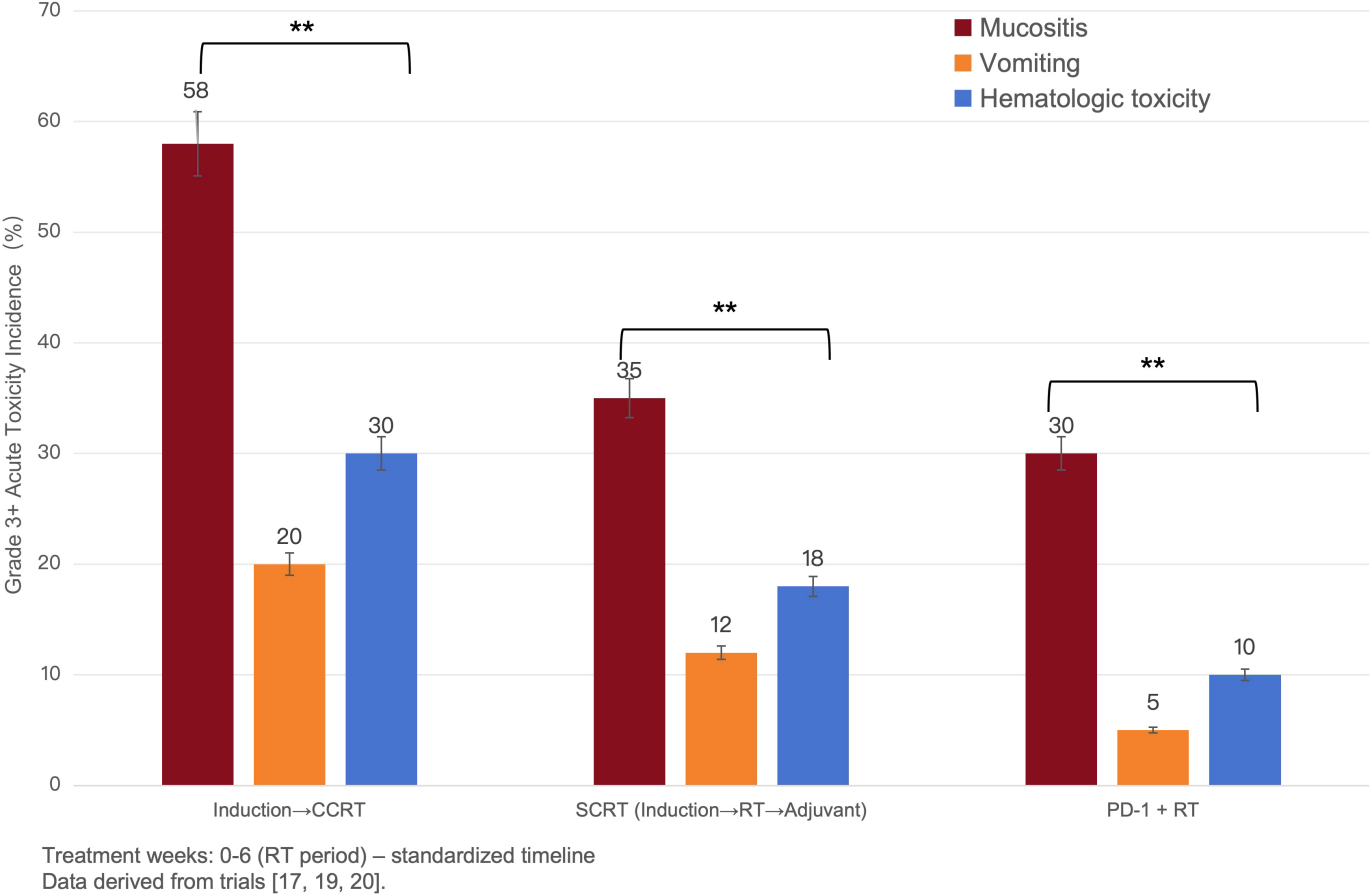
Comparison of acute toxicity profiles between novel temporal strategies and traditional concurrent chemoradiotherapy. The table summarizes data from Phase III trials: the SCRT regimen (Induction → RT → Adjuvant) reduces severe mucositis compared to induction-concurrent chemoradiotherapy (7). The PD-1 + RT regimen significantly reduces severe vomiting compared to traditional induction-concurrent chemoradiotherapy (8, 9). Time axes are standardized to weeks of treatment. Double asterisks (**) represent statistically significant intergroup differences in mucositis incidence.

The integration of immune checkpoint inhibitors has enabled more profound temporal strategy innovation. A key approach involves combining a PD-1 inhibitor with radiotherapy alone, omitting concurrent cisplatin. Phase II/III trials have shown this strategy can achieve non-inferior survival outcomes while dramatically reducing acute chemotherapy-related toxicities like severe vomiting and hematologic toxicity (8, 9). Landmark studies such as the JUPITER-02 and CONTINUUM trials have established the efficacy of immunotherapy in both metastatic and locoregionally advanced settings, paving the way for its use in de-intensification strategies (23, 24). Research continues to refine these combinations and explores their role even in resource-varied settings (25) (See **Figure 2**; **Table 1**).

### 3.3 Summary, controversies, and strategic reflections

Temporal innovations provide flexibility but introduce trade-offs:

- SCRT as an alternative, not a replacement — While SCRT reduces acute toxicity, it prolongs total treatment time, which may disadvantage patients with rapidly proliferating tumors. It is best suited for CCRT-intolerant patients.
- Immunotherapy shifts toxicity profiles — Reducing chemotherapy toxicity introduces immune-related adverse events (irAEs) like pneumonitis, colitis, and endocrinopathies, which require new diagnostic and management expertise globally (26).
- Economic and equity considerations — Immunotherapy costs can create “financial toxicity,” shifting burden to patients or payers. This raises health-system equity issues worldwide.
- Foundation and evolution of standards — The current paradigms are built upon the foundation of CCRT established by landmark trials (27, 28) and the subsequent demonstration of the benefit of adding induction chemotherapy (29). Temporal strategies represent an evolution seeking to optimize this balance.

## 4 Energy dimension strategy: the promise and reality gap of advanced technologies

### 4.1 Core concept: using Bragg peak physics for tissue protection

Intensity-modulated proton therapy (IMPT) leverages the Bragg Peak property: protons deposit most energy at a preset depth (the tumor), with minimal dose beyond it (30). Unlike photons, which exit through healthy tissue, protons can sharply reduce exposure to organs at risk, offering a potential physical advantage for “toxicity reduction.”

### 4.2 Key clinical evidence and global experience

While no Phase III survival trial for *de novo* NPC exists, extensive matched-pair analyses, systematic reviews, and Phase II data support IMPT’s dosimetric advantages (31, 32). Compared to advanced photon IMRT, IMPT consistently reduces mean dose to parotids, oral cavity, constrictors, cochlea, brainstem, and temporal lobes, with reductions commonly reaching 30-50% (11). Clinically, this translates to reports of lower acute toxicities and better patient-reported quality of life from leading proton centers worldwide (10). For challenging re-irradiation cases, IMPT is particularly valued for enabling safer salvage treatment (33) (see **Table 1**).

### 4.3 Challenges, positioning, and global value reflection

Proton therapy faces three universal challenges:

- Lack of Level I survival evidence — High costs, limited access, and patient preferences hinder the conduct of large-scale Phase III randomized controlled trials (RCTs). This leaves a gap in the highest level of comparative effectiveness evidence, which is crucial for widespread adoption and reimbursement.
- Health economics debate — Is investing in proton centers cost-effective compared to improving IMRT access, early detection, or supportive care? Systematic reviews highlight the significant economic burden, making this a critical global resource-allocation question (34).
- Technical and patient selection limits — IMPT is more sensitive to anatomical motion and setup error than photon therapy, requiring precise targeting. It should be positioned as a strategic tool for specific goals, such as protecting critical structures adjacent to the target or performing re-irradiation—not as a universal upgrade for all patients (35).

## 5 Integration challenges and future directions: toward systematic, individualized decisions

### 5.1 The clinical decision maze of multi-dimensional integration

Choosing among spatial, temporal, and energy strategies is complex and lacks direct comparative evidence. Current decisions rely heavily on physician experience, institutional resource access, guidelines, and patient values. The challenge is further compounded by the fact that these dimensions are not mutually exclusive and can be combined in various ways. For example, a patient might receive response-adapted target volumes (spatial) and immunotherapy-based de-escalation (temporal), with the potential addition of protons (energy) if available.

Future progress depends on moving towards integrative prediction systems. These systems would utilize multi-omics data and liquid biopsies (e.g., circulating EBV DNA) to model individual recurrence risk and organ-specific toxicity profiles (20, 36, 37). Artificial intelligence (AI) would be central to analyzing these complex datasets and guiding personalized strategy selection (38, 39).

### 5.2 Future directions: building a new system

**Figure 3:**
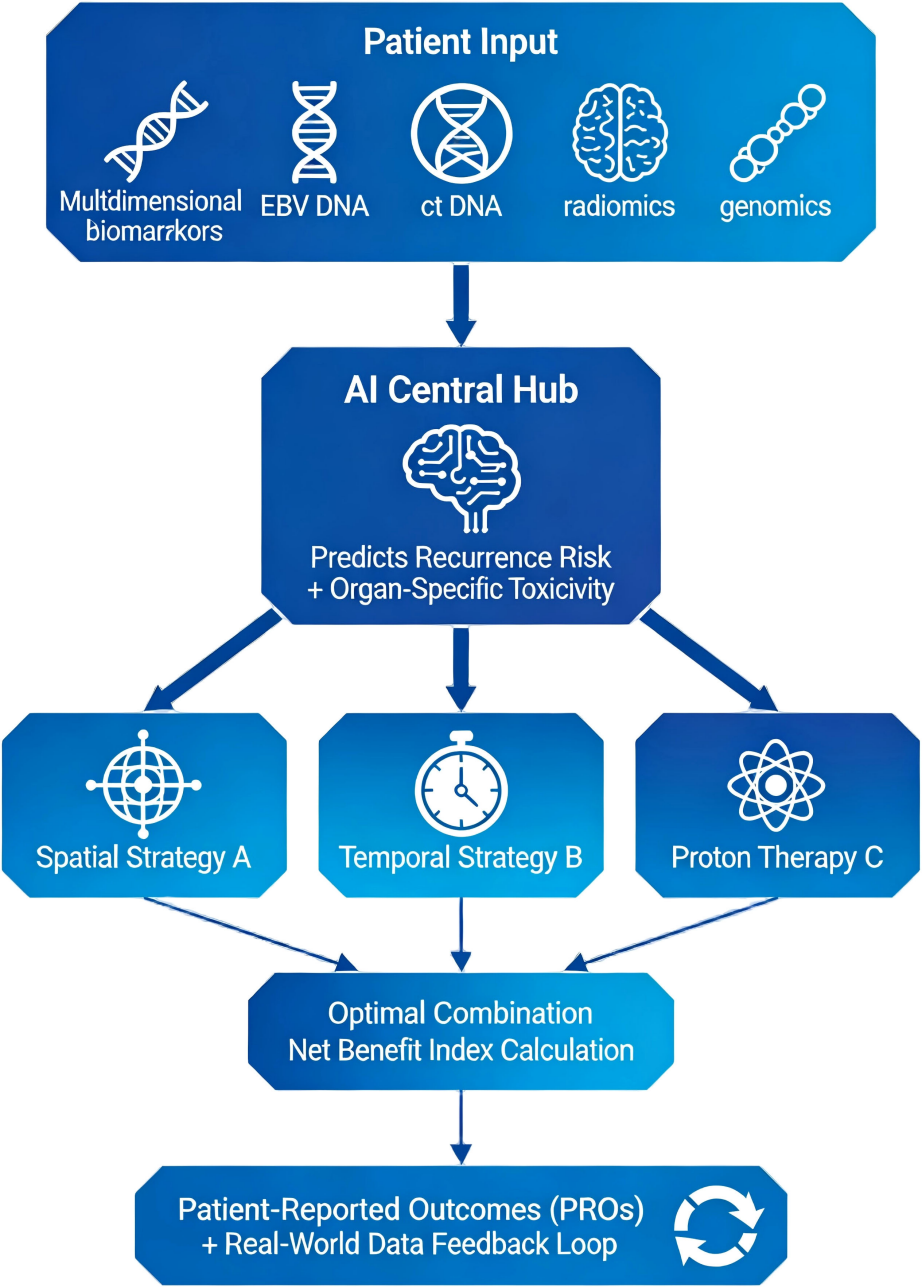
Schematic of a proposed AI-integrated decision-support system for personalized toxicity reduction strategy selection. Multi-omics data (e.g., genomics, radiomics from imaging, liquid biopsy for EBV DNA) and patient characteristics feed into an AI hub. The AI predicts individual risks of recurrence and specific toxicities, simulating outcomes for various combinations of spatial, temporal, and energy strategies. It then recommends an optimal, personalized treatment plan. Patient-Reported Outcomes (PROs) and real-world data enable the system to continuously learn and improve.

- Artificial Intelligence as a decision-support engine — AI’s role will extend beyond auto-contouring (40). It should evolve into a central hub that integrates multimodal data (imaging, genomics, proteomics, comorbidities, patient preferences), simulates efficacy-toxicity trade-offs for different strategy combinations, and recommends personalized, risk-adapted plans. This directly addresses the “clinical decision maze” by providing a quantitative, evidence-based synthesis of complex information (See **Figure 3**).
- Patient-Reported Outcomes (PROs) as core endpoints — The ultimate judgment of “toxicity reduction” lies with the patient. Future clinical trials must elevate PROs from secondary to co-primary or key secondary endpoints, alongside traditional survival metrics (41). This aligns treatment success directly with patient experience and quality of life (42).
- Molecular-targeted toxicity reduction — Beyond physical and temporal strategies, the future lies in molecular biology. This includes developing tumor-specific radiosensitizers and normal-tissue protectors, aiming for differential radioprotection at the molecular level (See **Figure 3**).

### 5.3 Global translation, validation, and equity challenges

Most evidence for the reviewed strategies comes from EBV-associated non-keratinizing NPC in high-incidence regions. While this subtype represents >95% of global cases, the effectiveness and optimal application of these strategies in other epidemiological contexts require validation through prospective international multi-center studies. Furthermore, the implementation of advanced strategies must be guided by considerations of global health equity to ensure benefits are widely accessible. This endeavor must be informed by and adhere to evolving international clinical practice guidelines (43, 44).

## 6 Conclusion

Having largely overcome the survival challenge, NPC treatment now focuses on toxicity reduction as a central goal. Leading research from high-incidence regions provides a transformative strategic blueprint built on the three-dimensional framework of spatial precision, temporal optimization, and energy advancement. True progress, however, lies not in pursuing any single technological pinnacle, but in building a globally integrated decision-science system. This system must combine high-quality evidence, predictive biomarkers, patient-centered outcomes, and prudent health-economic evaluation. The integration of AI offers a powerful pathway to navigate the “clinical decision maze,” synthesizing multidimensional data to guide personalized treatment. Facing the complexity of integration, biological uncertainty, and resource ethics, the global NPC community needs more open dialogue, data sharing, and equitable collaboration. Only through such concerted worldwide efforts can we ensure that every NPC patient not only survives but thrives with quality of life—thereby fully realizing the vision of moving from “disease cure” to “holistic recovery.”
